# Supplementary material for: Continuous 3D printing from one single droplet
Source: Nat Commun. 2020 Sep 17;11:4685. doi: 10.1038/s41467-020-18518-1 (PMC7499235; doi:10.1038/s41467-020-18518-1)
Supplement: Supplementary file 1 — Supplementary Information [file 41467_2020_18518_MOESM1_ESM.pdf]

SUPPLEMENTARY INFORMATION FOR

Continuous 3D printing from one single droplet

Zhang et al.

Correspondence to: wulei1989@iccas.ac.cn; ylsong@iccas.ac.cn

Supplementary Figures

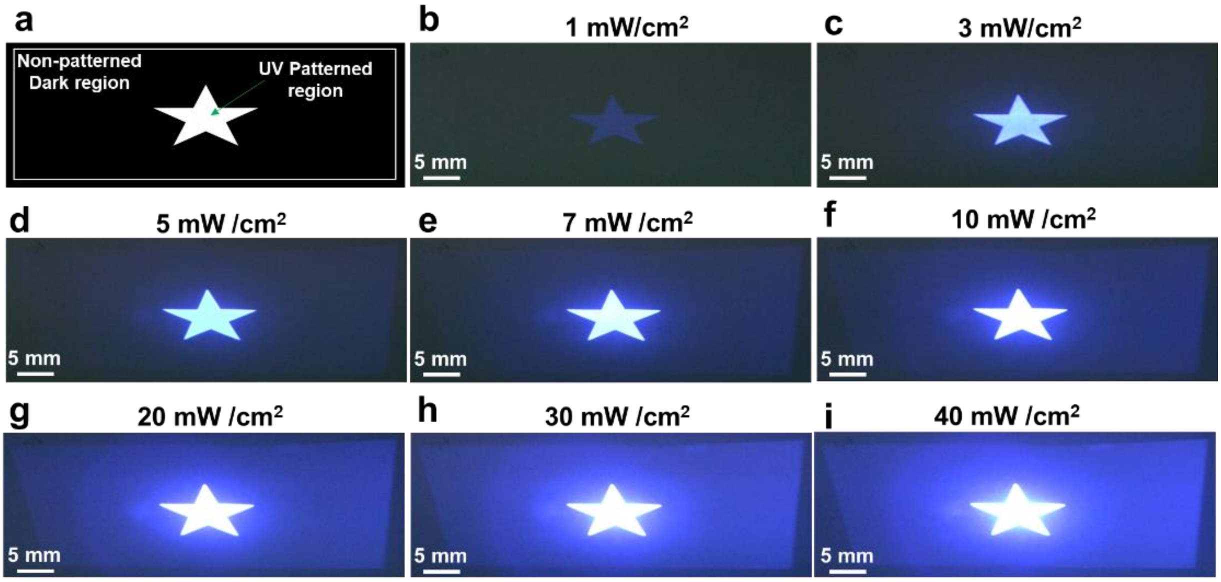

Supplementary Figure 1 | Afterglow of the 'dark' region of the UV projector under different light intensities. (**a**). Scheme of the patterned region and the non-patterned region of the UV pattern projected from the UV source. The star shape is the tested pattern and the white frame represents the breath of the UV projector. (**b**)-(**i**). Optical images of the UV pattern when projecting the pattern in (**a**) with different UV intensities (from 1 mW/cm^2 to 40 mW/cm^2). The images are captured with the camera settings (including the exposure time, magnification, photosensibility, and aperture, *et al.*) unchanged. The UV projector can provide a 405 nm pattern with the projection area of 51.6 mm × 32.2 mm. With the increasing of the UV intensity, the afterglow across the whole non-patterned dark region increases significantly, which finally may lead to the curing of the non-patterned dark region.

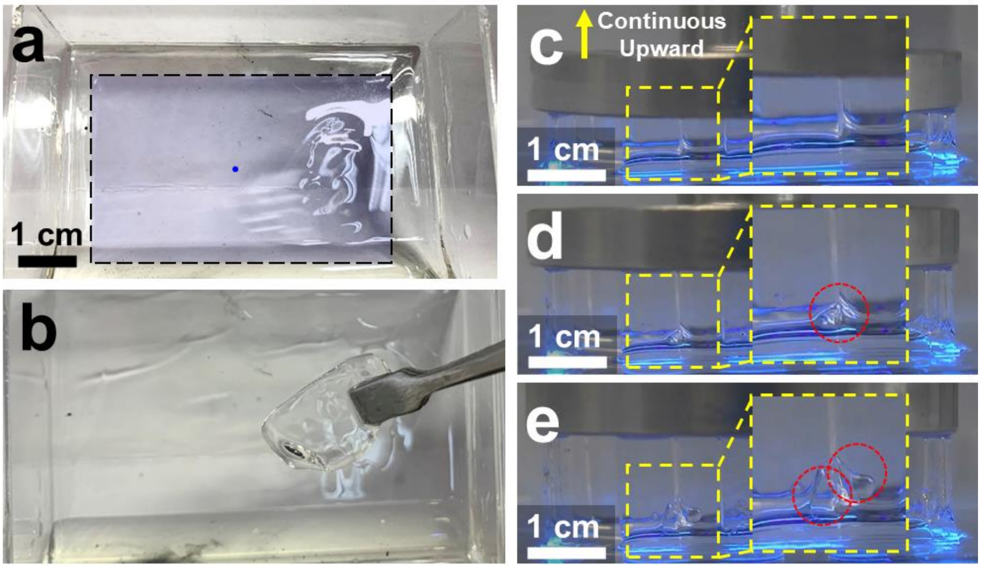

Supplementary Figure 2 | The unintentional curing of the 'dark' region during the vat polymerization process. (**a**). Optical image of the vat after continuous printing the typical pattern (1 mm round shape) characterized in the Figure 2 (UV intensity of 30 mW/cm^2 and printing speed of 100 μm/s). The blue dot and the square black dotted frame are scheme of the UV pattern and the breadth of the UV projector, respectively. (**b**). Optical image of the unintentionally cured resin. (**c**) - (**e**). Series of optical captures of the curing of the “dark” region which influences the printing precision. Insets are enlarged view of corresponding regions. Red circles indicate the cured part of the 'dark' region which connects to the patterned region.

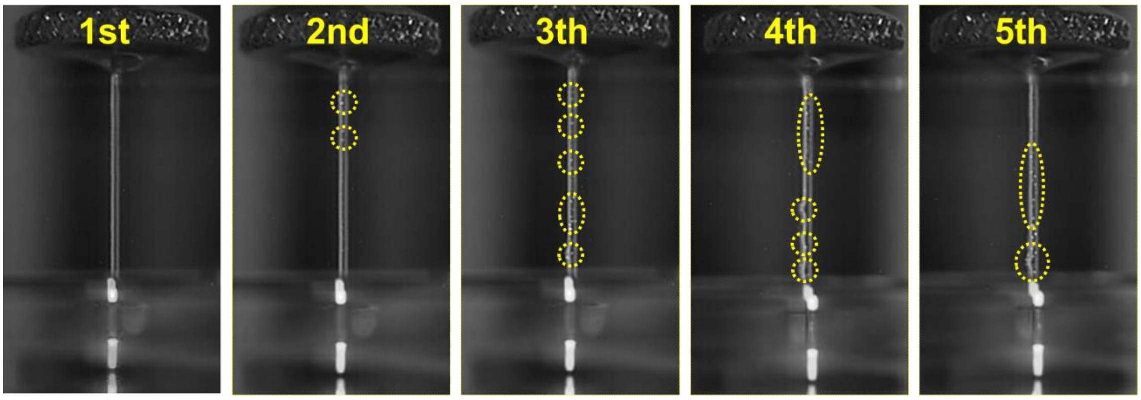

Supplementary Figure 3 | Optical captures of the cured structure after repeatedly using the same vat resin through vat polymerization. The experiment is conducted through repeatedly utilizing the same vat liquid resin at the same position with the same UV pattern, UV intensity and printing speed (50 mW/cm^2, 200 μm/s).

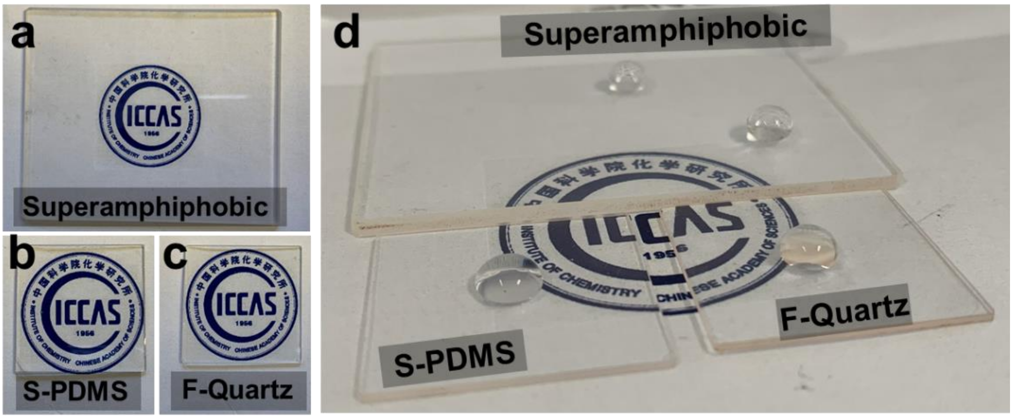

Supplementary Figure 4 | Optical characterization of different curing interfaces. (**a**), (**b**) and (**c**) are optical images of the superamphiphobic surface, the S-PDMS surface and the F-Quartz surface, respectively. (**d**). Optical image of the three surfaces with water droplets sitting on each surface.

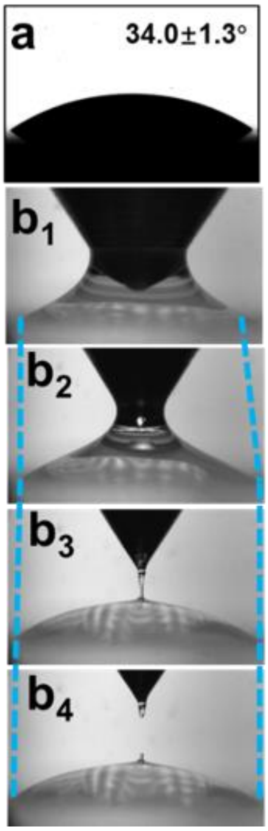

Supplementary Figure 5 | UV curing process on the quartz surface. (**a**) The contact angle of liquid resin on the quartz surface. (**b**$_1$)-(**b**$_4$) Optical images of the UV curing process on the quartz surface.

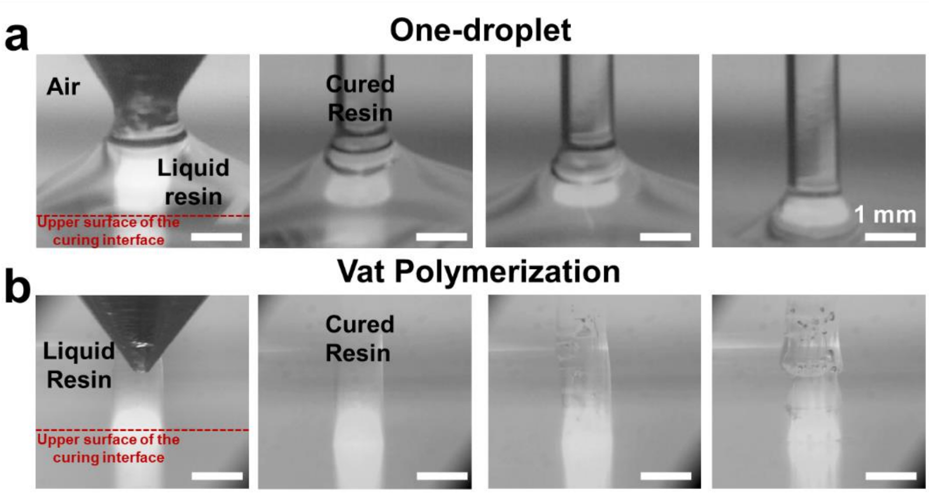

Supplementary Figure 6 | Comparison of the continuous printing process of the vat polymerization and the one-droplet 3D printing process (printing speed of 100 μm/s, 500 s duration for printing

structure of 5 cm high). (**a**). Series of optical captures of the one-droplet 3D printing process. (**b**). Series of optical captures of the vat polymerization process. Scale bars are 1 mm.

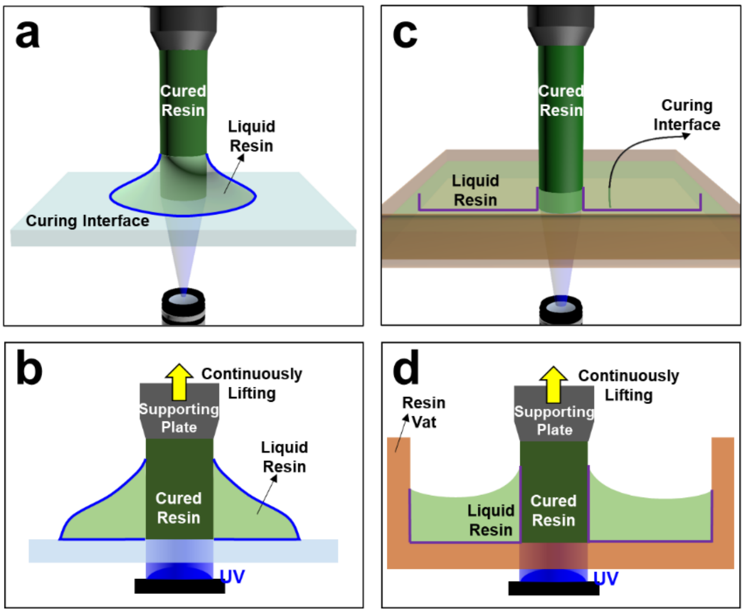

Supplementary Figure 7 | Comparison of the contact surface mobility of the one-droplet 3D printing and the vat polymerization process. (**a**). Scheme of the configuration of the one-droplet 3D printing process. (**b**). Scheme of the free contact surfaces during the one-droplet 3D printing process. Blue lines are the outlines of the free contact surfaces. (**c**). Scheme of the configuration of the vat polymerization process. (**d**). Scheme of the confined contact surfaces of the vat polymerization process. Purple lines are the confined contact surfaces.

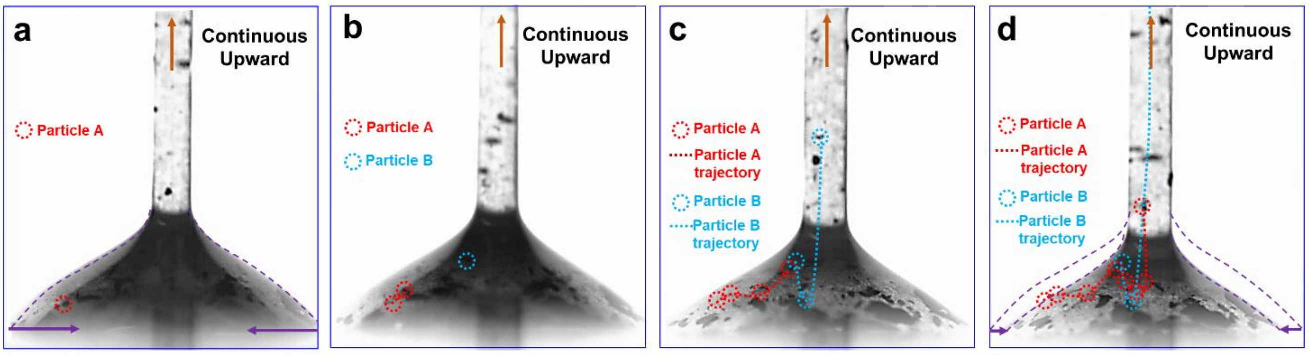

Supplementary Figure 8 | Optical tracking of the inner liquid flow during the continuous one-droplet 3D printing process through adding lightweight and black carbon nanofibers inside the liquid resin droplet. (**a**). The initial position of Particle A (red circle) in the liquid resin droplet. (**b**). The initial position of Particle B and part of the trajectory of particle A. Blue circle represent the initial position of Particle B. (**c**). The trajectories of particle A and particle B in the liquid resin and cured resin structure. Red and blue dot lines indicate the trajectories of particle A and particle B, respectively. Red dot lines indicate the trajectories of particle A and particle B, respectively. (**d**). The trajectories of particle A and particle B in the liquid resin and cured resin structure. Red and blue dot lines indicate the trajectories of particle A and particle B, respectively. Carbon nanofibers are selected as the additive, as their color is black for easy tracking, and their weight is light enough to flow along with the internal liquid flow easily. With the continuous lifting of the supporting plate, the inner droplet liquid can flow along the liquid resin-air interface upward to the cured structure-liquid resin interface (the trajectory of Particle A in Supplementary Figure 8a-c) driven by the adhesion of γ_1, or along the sidewall of the cured structure downward to the curing interface (the trajectory of Particle A in Supplementary Figure 8c-d and the trajectory of Particle B in Supplementary Figure 8c) driven by the adhesion of γ_3. The liquid resin inside the whole droplet is thus continuously moving, and the liquid resin is mixed uniformly during the continuous printing process.

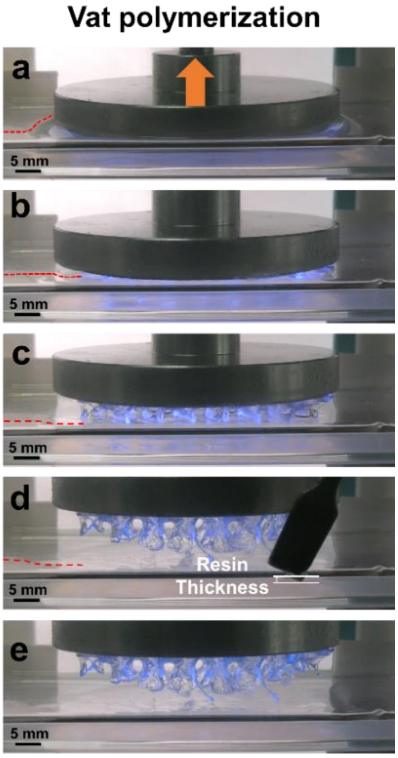

Supplementary Figure 9 | The unavailability of vat resin for curing even with a few printing layers high. (**a**)-(**e**). Optical captures of the vat polymerization process of the gyroid structure with the dimension in the x-y plane of 4.5 cm × 2.3 cm (5mW/cm^2, printing speed of 20 μm/s). The red lines in (**a**)-(**d**) are outlines of the upper surface of the vat resin.

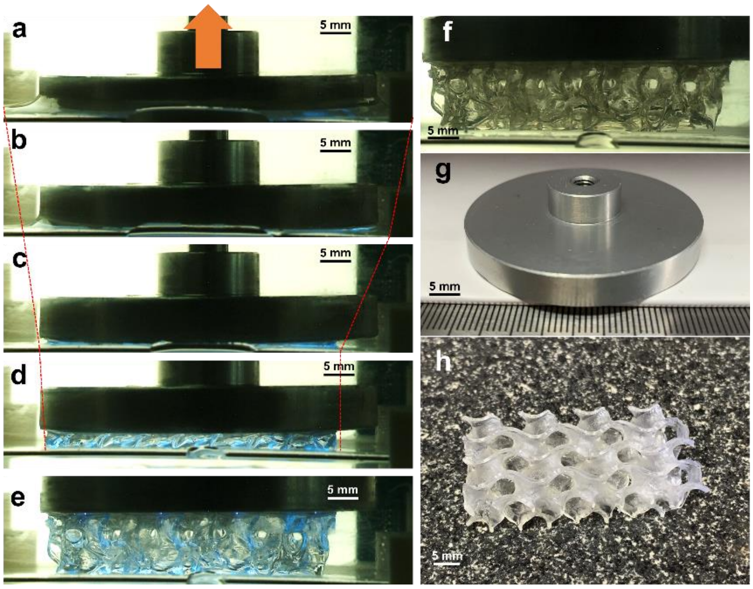

Supplementary Figure 10 | One-droplet 3D printing a gyroid structure. (**a**)-(**e**). Optical captures of the one-droplet printing process of the gyroid structure with the dimension in the x-y plane of 4.5 cm × 2.3 cm. (**f**). Optical capture of the printed structure after UV off. (**g**). Optical image of the supporting plate employed for printing the gyroid structure. (**h**). Optical image of the gyroid structure printed from one-droplet.

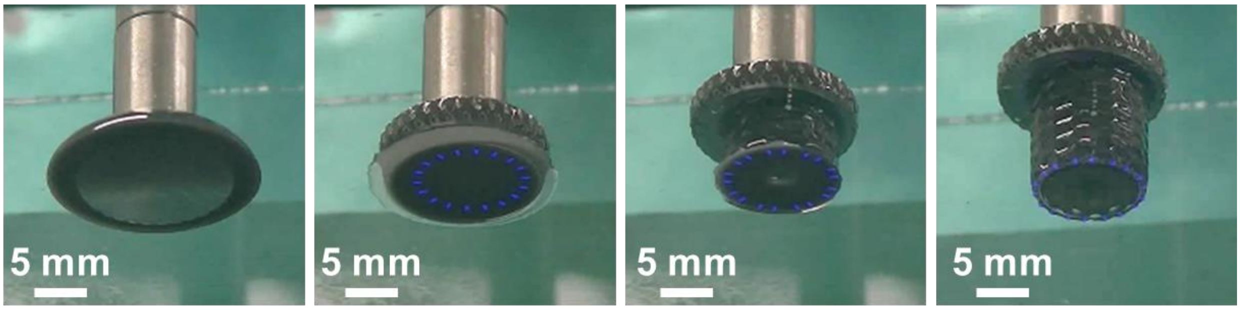

Supplementary Figure 11 | Optical captures of the one-droplet 3D printing process of a commercial flexible resin (Flexible Resin, Formlabs, America).

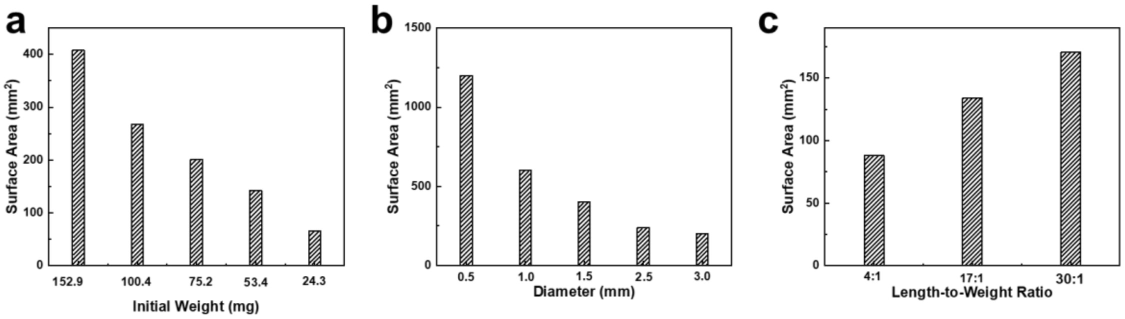

Supplementary Figure 12 | Comparison of the structure surface areas obtained under different experimental conditions. (**a**) The prepared structure surface area versus the initial weight (with the same projection pattern). (**b**) The prepared structure surface area versus the projection diameter (with the same initial weight). (**c**). The prepared structure surface area versus the L/W ratio (with the same projection area and the same initial weight).

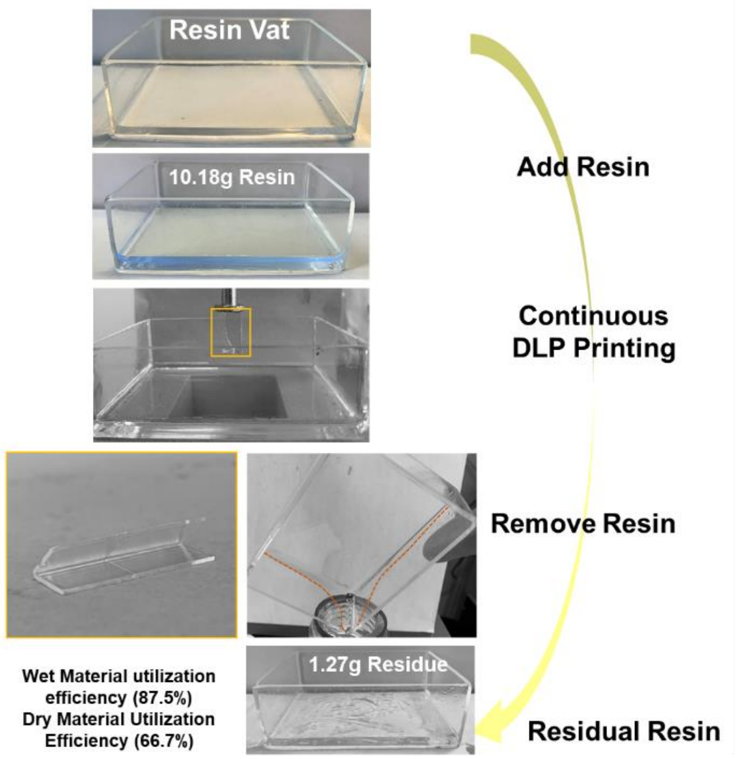

Supplementary Figure 13 | Characterization of the wet and net material utilization efficiencies in commercial 3D printing using a resin vat. The resin vat is a quartz box with dimensions of 60 mm ×

90 mm × 45 mm and a wall thickness of 2 mm. The bottom of the quartz surface is covered with a PDMS film.

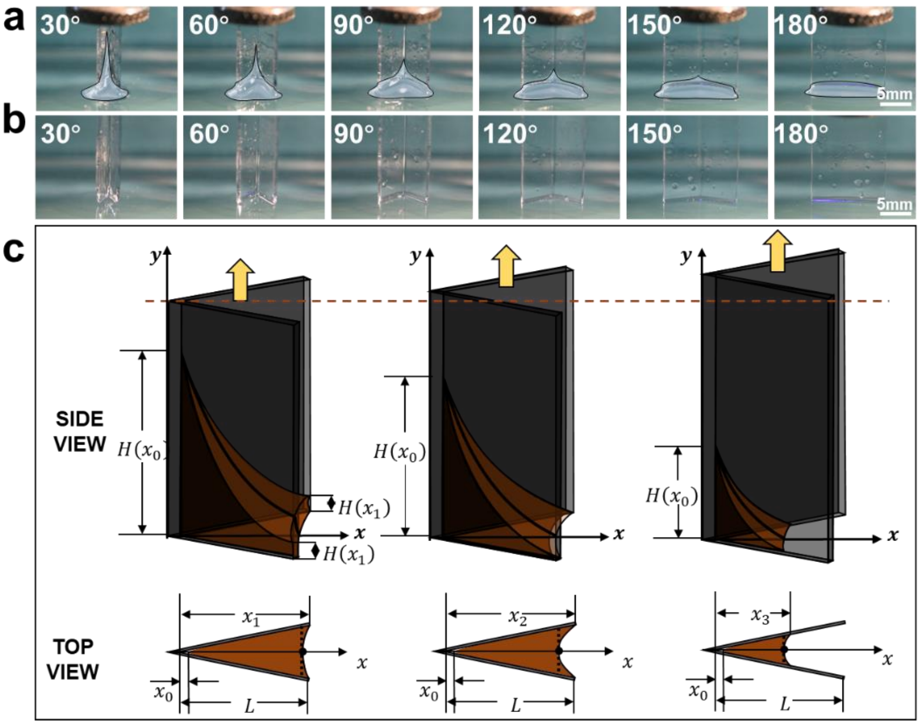

Supplementary Figure 14 | Scheme of the 3D structure influenced the process. (**a**)-(**b**) Optical images of the liquid resin rising height during the one-droplet printing process of V-grooved structures of different included angles (L/W ratio of 17:1). (**a**) and (**b**) Optical images of the intermediate state and the final state of the one-droplet 3D printing process. (**c**) Scheme of the liquid resin dewetting process on the cured V-grooved structure under the action of the capillary force and the definition of the *x*-*y* plane.

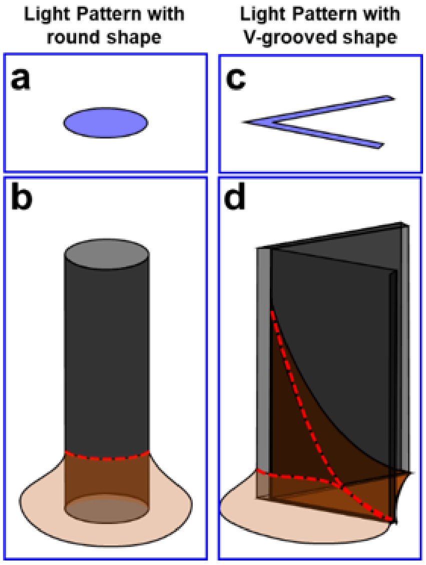

Supplementary Figure 15 | Scheme of the contact line morphology on the cured 3D structure for different UV projection patterns. (**a**) Scheme of the UV pattern with a round shape. (**b**) Scheme of the contact line morphology on the cured 3D structure for the UV projection pattern with a round shape. (**c**) Scheme of the UV pattern with a V-grooved shape. (**d**) Scheme of the contact line morphology on the cured 3D structure for the UV projection pattern with a V-grooved shape. The red dotted line indicates the contact line of liquid resin on the cured structure.

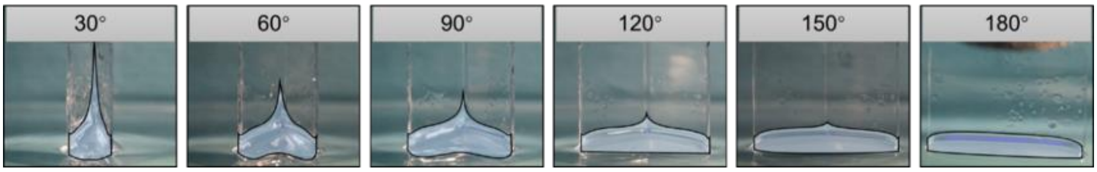

Supplementary Figure 16 | Optical images of the rising height of liquid resin on the cured structures with different intersection angles.

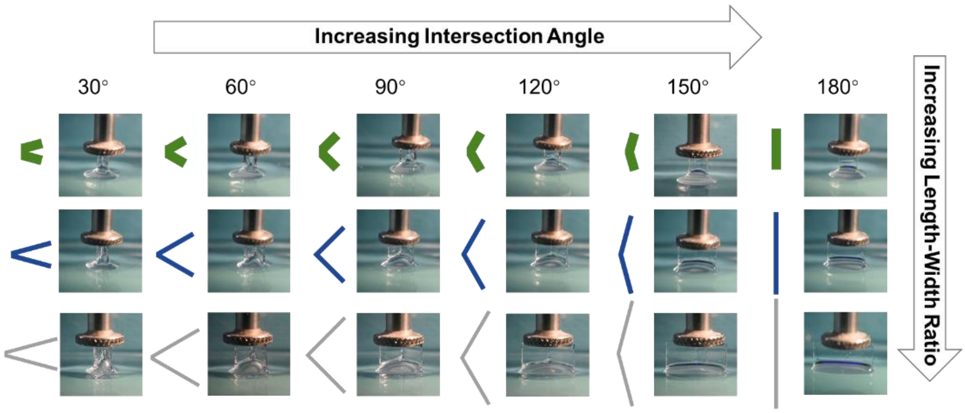

Supplementary Figure 17 | Optical images of the 3D distribution of liquid resin on the cured structures during the printing process under different conditions.

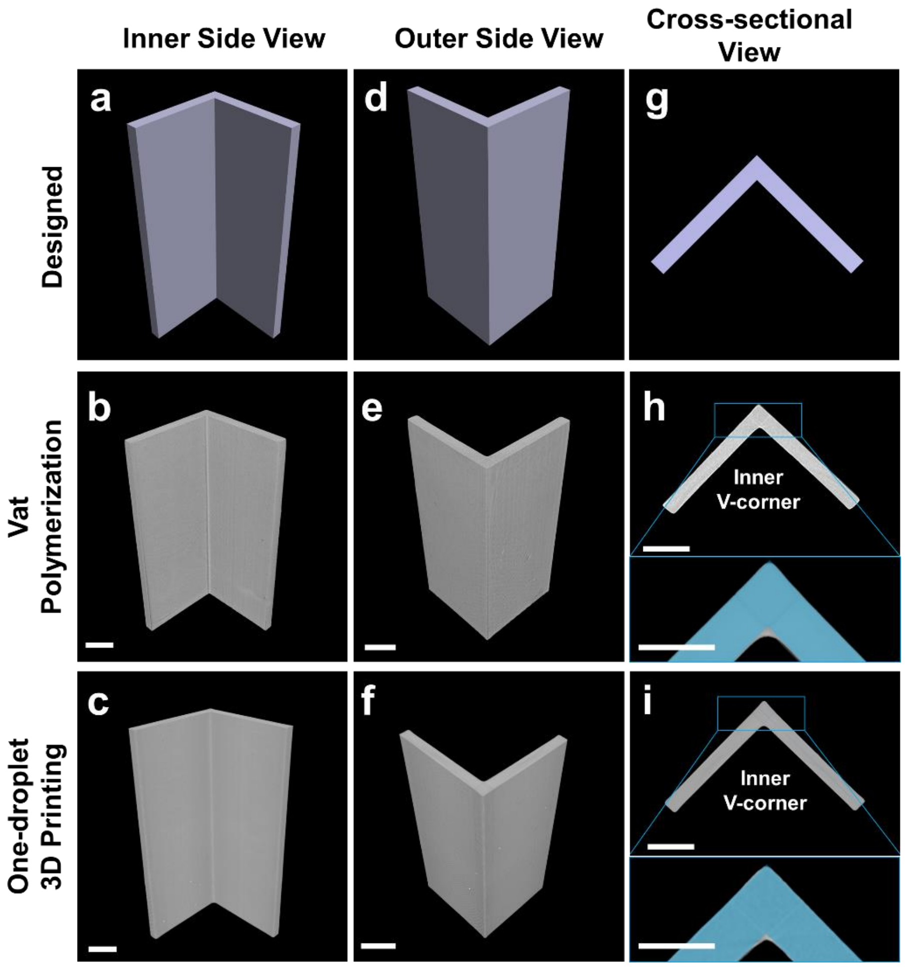

Supplementary Figure 18 | Micro-CT characterization of the V-grooved structures prepared from vat polymerization and one-droplet 3D printing. (**a**), (**b**) and (**c**) are inner side view of the designed, Micro-

CT image of V-grooved structure prepared from vat polymerization and Micro-CT image of V-grooved structure prepared from one-droplet 3D printing, respectively. (**d**), (**e**) and (**f**) are outer side view of the designed, Micro-CT image of V-grooved structure prepared from vat polymerization and Micro-CT image of V-grooved structure prepared from one-droplet 3D printing, respectively. (**g**), (**h**) and (**i**) are cross-sectional view of the designed, Micro-CT image of V-grooved structure prepared from vat polymerization and Micro-CT image of V-grooved structure prepared from one-droplet 3D printing, respectively. The blue V patterns in **h** and **i** are designed V patterns. Scale bars are 2 mm.

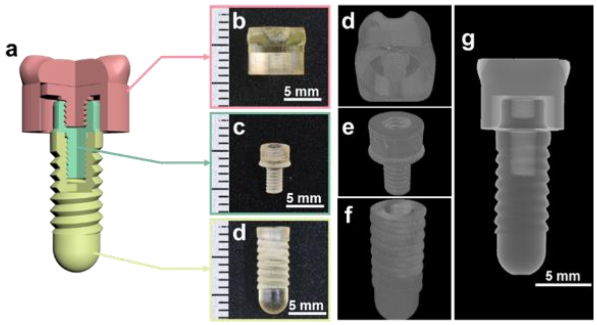

Supplementary Figure 19 | Scheme and characterization of the one-droplet 3D printing process of curing a resin droplet into the desired model. (**a**) Scheme of a 3D structure composed of three assembled parts. (**b, c**) Optical images of the three parts fabricated with the one-droplet 3D printing process. (**d, e, f**) Micro-CT characterization of the three parts. (**g**) X-ray transmission image of the three parts after assembly.

Supplementary Methods

I. Calculation of polar and dispersive components of different curing interfaces: The OWRK (Owens, Wendt, Rabel and Kaelble) method. For a solid-liquid interface, the work of adhesion can be expressed as follows:

$$W_a = \gamma_S + \gamma_L - \gamma_{SL} \tag{1}$$

The adhesion can be divided into polar and dispersive parts. As proposed by Fowkes, the polar and dispersive interfacial attractions can be treated independently, and the polar-dispersive interactions can be neglected.

$$W_a = W_a^d + W_a^p = 2(\sqrt{\gamma_S^d \gamma_L^d} + \sqrt{\gamma_S^p \gamma_L^p}) \tag{2}$$

According to Young's equation, when contact angle θ > 0:

$$\gamma_S = \gamma_{SL} + \gamma_L \cos\theta \tag{3}$$

Combining these equations:

$$\frac{\gamma_L(1+\cos\theta)}{2\sqrt{\gamma_L^d}} = \sqrt{\gamma_S^d} + \sqrt{\gamma_S^p}\frac{\sqrt{\gamma_L^p}}{\sqrt{\gamma_L^d}} \tag{4}$$

The polar and dispersive components of the solvent are known; thus, plotting the left side of Supplementary Equation 4 against $\sqrt{\gamma_L^p}/\sqrt{\gamma_L^d}$ will theoretically produce a line of data points. Then, $\sqrt{\gamma_S^d}$ and $\sqrt{\gamma_S^p}$ can be calculated using the data of more than two liquids with given surface tension components. Taking the quartz surface as an example, the estimated results are γ_S = 42.5 mN/m, γ_S^d = 19.52 mN/m and γ_S^p = 22.93 mN/m, as shown in Supplementary Figure 20. The polar and dispersive components of other interfaces used in this manuscript and the cured resin are calculated based on Supplementary Table 1 and Supplementary Table 2 and listed in Supplementary Table 3.

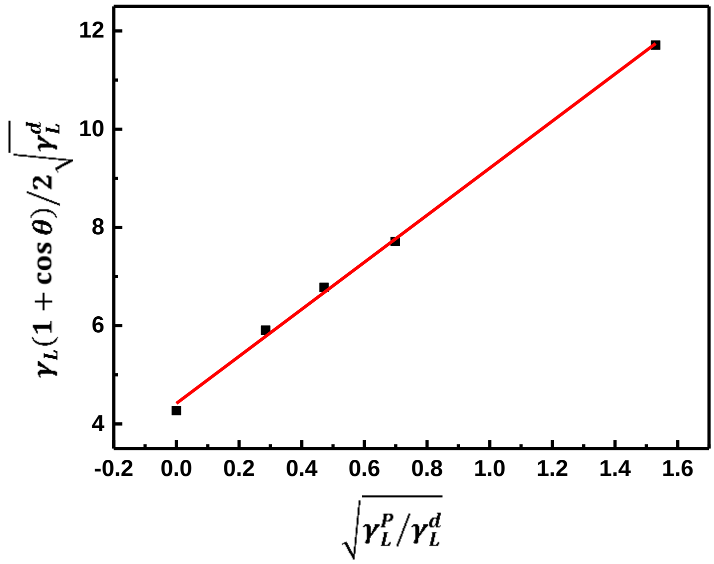

Supplementary Figure 20 | Fitted curve of the surface tension of the quartz surface obtained with the OWRK method. Five test liquids are used to increase the accuracy of the results: water, ethylene glycol, dimethyl sulfoxide, ethylene dichloride, and n-hexane. The estimated results are γ_S = 42.5 mN/m, γ_S^d = 19.52 mN/m and γ_S^p = 22.93 mN/m. Error bars originate from the result from errors in the contact angle measurements.

Supplementary Table 1 | Dispersive and polar components of surface energy for the probe liquids.

Liquid	Abbreviation	SE ($\sqrt{\gamma_L}$) mJ/m^2	DSE ($\sqrt{\gamma_L^d}$) mJ/m^2	PSE ($\sqrt{\gamma_L^p}$) mJ/m^2
Water	H_2O	72.8	21.8	51[1]
Ethylene glycol	EG	48.8	32.8	16[2]
Dimethyl Sulfoxide	DMSO	44	36	8[1]
Ethylene Dichloride	EDC	33.3	30.8	2.5[1]
N-hexane	NH	18.4	18.4	0[1]

Note: SE: surface energy, DSE: dispersive component of surface energy, PSE: polar component of surface energy.

Supplementary Table 2 | Measured contact angles of probe liquids on the used curing interfaces and

the cured resin.

Liquid	Contact angle (°)						
	Quartz	F-Quartz	S-PDMS	Superamphiphobic	Cured Resin 1	Cured Resin 2	Cured Resin 3
H_2O	59.9 ± 2.3	106.2 ± 3.3	101.9 ± 1.4	160.1 ± 0.4	72.8±4.8	66.5±3.9	98.0±4.4
EG	35.9 ± 1.7	93.2 ± 2.3	83.1 ± 1.6	157.8 ± 1.8	51.5±4.1	39.9±2.2	85.8±4.7
DMSO	31.9 ± 1.4	86.9 ± 1.9	73.6 ± 2.1	153.5 ± 1.2	23.8±4.1	39.5±1.7	58.4±3.2
EDC	14.3 ± 0.6	61.1 ± 0.5	35.8 ± 1.1	149.8 ± 1.5	24.6±5.6	32.6±1.3	41.8±6.6
NH	7.4 ± 1.2	32.8 ± 4.0	< 5	145.8 ± 3.4	7.4±1.3	8.9±0.6	3.2±0.8

Note: Resin 1, Resin 2 and Resin 3 are the resin used in Figure 1-3, the resin used in Figure 4 and the commercial flexible resin, respectively.

Supplementary Table 3 | Components of surface energies for the used curing interfaces and the cured resin.

Solid	SE ($\sqrt{\gamma_L}$) mJ/m^2	DSE ($\sqrt{\gamma_L^d}$) mJ/m^2	PSE ($\sqrt{\gamma_L^p}$) mJ/m^2
Quartz	22.9	19.5	42.5
F-Quartz	1.1	14.1	15.2
S-PDMS	3.9	17.4	21.3
Superamphiphobic	2.63E-03	3.91E-02	4.17E-02
Cured Resin 1	29.0	21.4	7.6
Cured Resin 2	36.7	18.3	18.4
Cured Resin 3	21.8	20.0	1.8

Note: SE: surface energy, DSE: dispersive component of surface energy, PSE: polar component of surface energy. Resin 1, Resin 2, and Resin 3 are the resin used in Figure 1-3, the resin used in Figure 4, and the commercial flexible resin, respectively.

<u>II. Calculation of polar and dispersive component of the three used liquid resins.</u>

In order to investigate the polar and dispersive surface tension of the liquid resin, we use the standard

surface method. The standard reference surface for two-component liquid surface tension determination is poly(tetrafluoroethylene) (PTFE). Pure untreated PTFE is assumed to have a surface energy of 18.0 mJ/m^2, and is assumed to have a surface energy of 18.0 mJ/m^2, and is assumed to be capable of no polar type interactions. In other words, $\gamma_L = \gamma_S^d$ =18.0 mJ/m^2 for PTFE, and γ_S^p =0 mJ/m^2 for PTFE. Substituting these values into the primary Owens/Wendt equation, followed by rearrangement yields:

$$\gamma_L^d = \gamma_L^2(\cos\theta_{PTFE} + 1)^2/72 \quad (5)$$

where θ_{PTFE} is the contact angle measured between PTFE and the probe liquid.

Therefore, the dispersive surface tension component (γ_L^d) can be determined for any liquid for which the overall surface tension (γ_L) is known, simply by measuring the contact angle between the liquid resin and the PTFE and using the Supplementary Equation 5. The polar surface energy component for the liquid is then determined by difference ($\gamma_L^d = \gamma_L - \gamma_L^d$). A high-sensitivity microelectro-mechanical balance system measures the overall surface tension of liquid.

For liquid Resin 1, the surface tension is 31.1 ± 1.2 mJ/m^2, the contact angle of polyacrylate liquid resin on PTFE is 62.2 ± 2.5°. Thus, according to Supplementary Equation 5, the dispersive component of the liquid resin γ_L^d is 25.3 mN/m, the polar component of the liquid resin γ_L^p is 5.8 mN/m.

For Resin 2, the surface tension is 35.8 ± 0.6 mJ/m^2, the contact angle of polyacrylate liquid resin on PTFE is 66.5 ± 4.3°. Thus, according to Supplementary Equation 5, the dispersive component of the liquid resin γ_L^d is 30.5 mN/m, the polar component of the liquid resin γ_L^p is 5.3 mN/m.

For Resin 3, the surface tension is 32.07 ± 0.8 mJ/m^2, the contact angle of polyacrylate liquid resin on PTFE is 59.6 ± 5.3°. Thus, according to Supplementary Equation 5, the dispersive component of the liquid resin γ_L^d is 28.3 mN/m, the polar component of the liquid resin γ_L^p is 3.8 mN/m. According to Supplementary Equation 2, the liquid resin-solid surface adhesion (including different curing interfaces) can be calculated and listed in Table 1 and Supplementary Table 5.

Supplementary Table 4 | Liquid Solid adhesion among different resin systems and curing interfaces.

Solid	Resin 1 - Solid Adhesion (mJ/m^2)	Resin 2 - Solid Adhesion (mJ/m^2)	Resin 3 - Solid Adhesion (mJ/m2)
Quartz	69.4	70.9	65.6
F-Quartz	48.8	46.3	44
S-PDMS	56.9	54.9	51.8
Superamphiphobic	3.7	4.1	3.9
Cured Resin 1	59.4	-	-
Cured Resin 2	-	67.1	-
Cured Resin 3	-	-	52.6

Note: SE: surface energy, DST: dispersive component of surface energy, PST: polar component of surface energy. Resin 1, Resin 2, and Resin 3 are the resin used in Figure 1-3, the resin used in Figure 4, and the commercial flexible resin, respectively.

Supplementary Table 5 | The summary of surface tensions and interfacial adhesions of different resin systems on the S-PDMS curing interface.

Resin	Surface Tension (mN/m)	Interfacial Adhesion (mJ/m^2)		
		Cured Resin /Curing Interface (γ_2)	Liquid Resin /Curing Interface (γ_3)	Liquid Resin /Cured Resin (γ_1)
Resin 1	31.1 ± 1.2	1.8 ± 0.3	56.9 ± 4.3	59.4 ± 2.9
Resin 2	35.8 ± 0.6	0.9 ± 0.5	54.9 ± 0.4	67.1 ± 0.8
Resin 3	32.1 ± 0.8	0.3 ± 0.2	51.8 ± 0.4	52.6 ± 0.7

Note: Resin 1, Resin 2, and Resin 3 are the resin used in Figure 1-3, the resin used in Figure 4, and the commercial flexible resin, respectively.

III. Derivation of the dragging force of liquid resin on the cured structure induced by the capillary rise:

The capillary rise height profile $h(x)$ along the symmetric surface of the V-grooved structure at a distance of x can be expressed as:

$$h(x) = \frac{2\gamma cos\theta}{\rho\alpha g x} \quad (6)$$

where γ is the surface tension of the liquid resin, θ is the contact angle of the liquid resin on the cured resin, ρ is the density of the liquid resin, α is the intersection angle of the V-grooved structure, g is the gravitational acceleration, and x is the selected position on the x axis.

The contact area between the liquid resin and cured structure S can be expressed as:

$$S = \int_{x_0}^{x_1} h(x)\, dx = \int_{x_0}^{x_1} \frac{2\gamma cos\theta}{\alpha\rho g} dx = \frac{2\gamma\cos\theta}{\alpha\rho g} \ln\frac{x_1}{x_0} \quad (7)$$

where x_1 and x_0 are two positions, as shown in Supplementary Figure 14c.

The work of adhesion of the liquid contact line on the cured structure W can be expressed as:

$$W = 2 \cdot S \cdot (\gamma - \gamma_S + \gamma_{ls}) \quad (8)$$

where γ_S is the surface tension of the cured resin, γ_{ls} is the interfacial tension between the liquid resin and the cured resin, and γ is the surface tension of the liquid resin.

According to Young's equation:

$$\cos\theta = \frac{\gamma_S - \gamma_{ls}}{\gamma} \quad (9)$$

W can be derived as:

$$W = 2 \cdot S \cdot \gamma \cdot (1 - \cos\theta) = \frac{4\gamma\cos\theta(1-\cos\theta)}{\alpha\rho g} \ln\frac{x_1}{x_0} \quad (10)$$

Thus, the force of the liquid resin contact line on the cured structure can be expressed as:

$$F_A = \frac{W}{vt} = \frac{1}{\alpha} \cdot \frac{4\gamma \cos\theta (1-\cos\theta)}{\rho g v t} \ln \frac{x_1}{x_0} \tag{11}$$

where v is the moving velocity of the supporting plate, and t is the time needed for printing the entire structure.

Supplementary References

[1] Van Oss, C. J. Interfacial Forces in Aqueous Media, 2nd ed.; Taylor & Francis: New York, 2006

[2] Mittal, K. L., Ed. Contact Angle, Wettability and Adhesion; VSP: Utrecht, The Netherlands, 1993.
