# Supplementary material for: Continuous 3D printing from one single droplet
Source: Nat Commun. 2020 Sep 17;11:4685. doi: 10.1038/s41467-020-18518-1 (PMC7499235; doi:10.1038/s41467-020-18518-1)
Supplement: Supplementary file 3 — Description of Additional Supplementary Files [file 41467_2020_18518_MOESM3_ESM.docx]

File Name: Supplementary Movie 1

Description: Real-time monitoring of one-droplet 3D printing process on different curing interfaces.

File Name: Supplementary Movie 2

Description: Comparison of the one-droplet 3D printing process and the vat polymerization process.

File Name: Supplementary Movie 3

Description: Real-time tracking of the inner liquid flow inside the droplet during the continuous one-droplet 3D printing process.

File Name: Supplementary Movie 4

Description: Real-time monitoring of a liquid droplet transfers to a tooth structure.
